# Out of field scatter from electron applicator in modern linear accelerators

**DOI:** 10.1002/acm2.14265

**Published:** 2024-02-09

**Authors:** Bishwambhar Sengupta, Greg DeFillippo, James J. Sohn, Poonam Yadav, Indra J. Das

**Affiliations:** ^1^ Department of Radiation Oncology Northwest Memorial Hospital Northwestern University Feinberg School of Medicine Chicago Illinois USA

**Keywords:** applicator design, electron beam, normal tissue dose, outside dose, scatter

## Abstract

**Background:**

Electron out‐of‐field scatter is generally not given importance mainly in electron fields. However, this is important when applicator down and boost treatments are given usually at an angle from the central axis. The electron scatter dose is found to be far away from the central axis which could be easily ignored.

**Purpose:**

This study aims to investigate the out‐of‐field radiation doses from electron applicators and their effects on clinical treatment. By identifying the parameters that contribute to out‐of‐field doses and to explore potential strategies for reducing these doses in order to improve patient outcomes from modern machines.

**Methods:**

Measurements were performed in water phantom using electron diode for modern Elekta and Varian machines. Dose profiles were acquired at surface and d_max_ with 0° and 90° collimation angle. Various gantry angles were also studied for some data with IC Profiler. The profiles were normalized with respect to the central axis dose.

**Results:**

The scatter dose peaks were found at a distance between 11 and 28 cm from the central axis on all machines. However, the peak shifts to 15 cm at 90° collimator when beam is tilted. The position and intensity of the dose varies with depth, collimator, and gantry angles for both Elekta and Varian machines. Due to clearance issues more gantry angles were studied for Elekta applicator compared to Varian. In general, Varian TrueBeam has a lower scatter that Elekta Infinity. The 90° collimator angle has a higher scatter compared to zero degree for both machines.

**Conclusions:**

There are clinically significant peripheral doses around 3% of the central axis dose from the electron applicator. Elekta has a slightly higher scatter (3%) than Varian (2%) that peaks at 25 cm which is clinically important but often overlooked.

## 1 INTRODUCTION

External beam radiation therapy is used for the management of cancer in both palliative and curative cases^1^ with up to 824,000 cancer patients receiving radiation therapy as a form of treatment annually in the United States.^2^ Electron beams are preferred in external beam radiation therapy for superficial targets and post‐surgical scars in many disease sites such as breast, head, and neck due to their rapid dose fall‐off and relatively high surface dose. Electron scattering has been discussed in detail that provides bedrock for modern electron beam interaction.^3^ To provide uniform dose in the treatment field, Bjärngard et al.^4^ provided a method for designing the electron applicator. In the modern era electron applicators are designed to provide optimum beam flatness based on jaw opening.^5^ The applicator designs from various vendors (Sagittaire, Saturne, Mitsubishi, Siemens, Elekta, and Varian)^6^, ^7^, ^8^, ^9^, ^10^, ^11^, ^12^, ^13^ are relatively similar except for the optimization of the vane, stopping plates, and jaw size. Pennington et al.^7^ provided a new design to improve the off‐axis scatter. Electron applicators are attached to the gantry head to stop large‐angle scatter electrons and provide a uniform dose within 80% of the field width. However, still, electron scatter could lead to off‐axis dose with undesirable comorbidities^14^ with some cosmetic effect that affects patient quality‐of‐life. Wen et al.^8^ provided a clinical case of significant alopecia associated with electron scatter.

The scatter dose from the applicator has been studied for Siemens, Elekta, and Varian machines^13^, ^15^, ^16^, ^17^ and found to be variable. For example, Chow et al.^18^ found that the maximum scatter dose in the Varian machine is 1.6% (for 4 MeV) at a 12 cm distance from the central axis. On the contrary, Yeboah et al.^16^ found 16% scatter dose at 28 cm from the central axis for 18 MeV for the Siemens machine. The magnitude of the peripheral dose is smaller for lower energies. In the case of megavoltage electron beams, an electron applicator is used to minimize the out‐of‐field dose to the patient. Peripheral dose from electron applicators has been extensively reported in the literature.^7^, ^9^, ^10^, ^15^, ^19^, ^20^ However, most of these studies were performed on relatively older machines such as Siemens and older Elekta Linacs. Additionally, previous works reported peripheral beam data at a gantry angle of zero degrees along the vertical axis, ignoring the common clinical setups that use oblique beams.

Modern electron applicators are designed to reduce scatter dose; however, they have different lateral side openings when viewed in 0° and 90° collimator angles. The impact of collimator angles has not been studied either for older or modern machines. In the current study, we measured electron scatter from Varian and Elekta linear accelerators to analyze the effect of gantry angle, collimator angle, beam energy, and depth of treatment. Method of mitigating effect of electron scatter for clinical cases is also provided.

**Novelty & Significance**

Electron beam therapy is an integral part of radiation therapy. However, out‐of‐field electron scatter from the applicator is not fully understood for the modern accelerators. Differences in out‐of‐field dose have been observed between Elekta and Varian linear accelerators with different applicator designs. The magnitude of the peripheral dose is relatively small but not negligible. There are significant peaks observed far away (11–28 cm) from the central axis that could easily be missed in clinical practice. The peaks are dependent on the machine and gantry angles and should be considered for normal tissue exposure.

## 2 MATERIALS & METHODS

**FIGURE 1:**
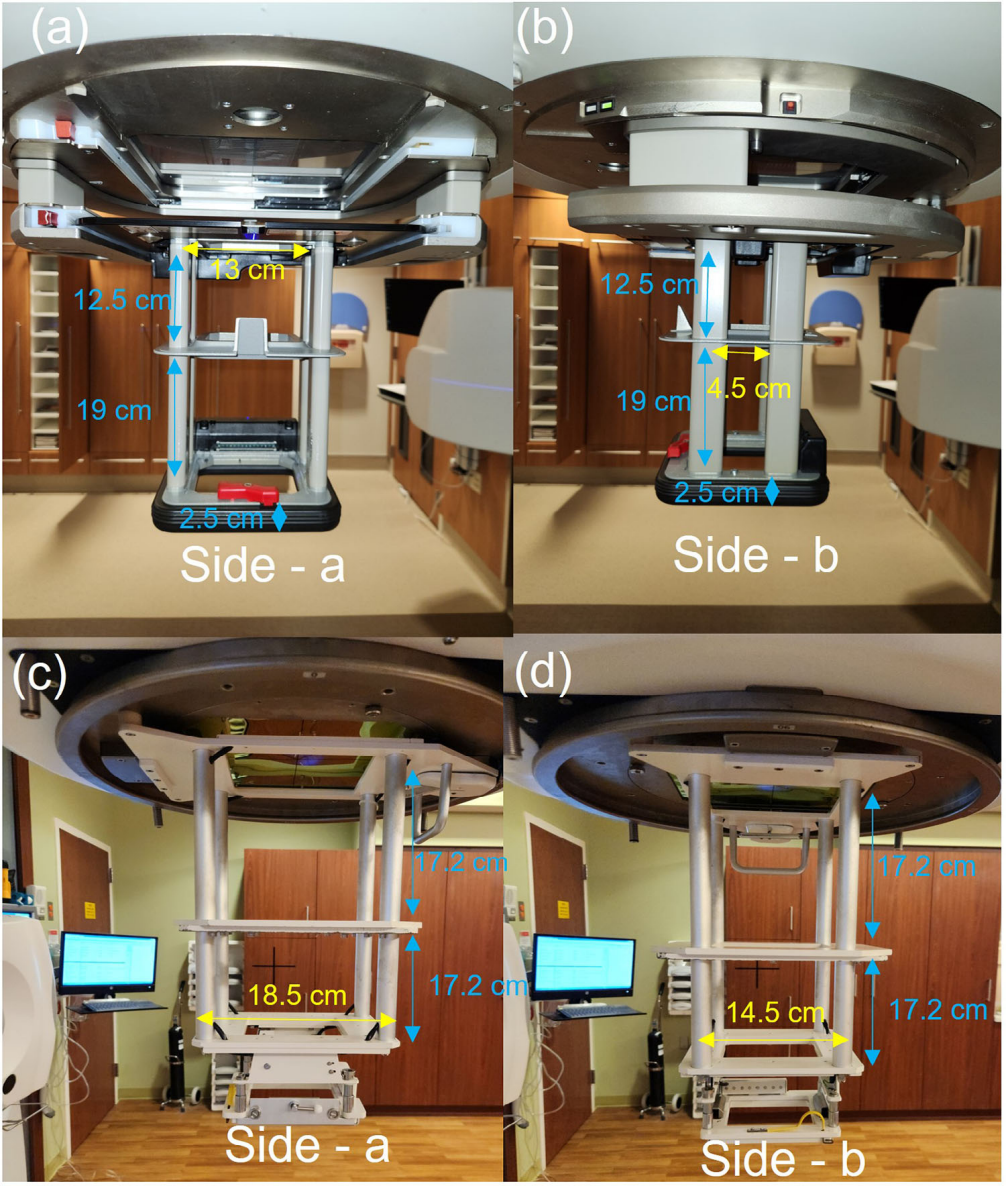
The construction of Varian (a, b) and Elekta (c, d) 10 × 10 electron applicators in side‐a and side‐b orientation.

Radiation dose outside the electron field was measured for electron beams on two linear accelerators: Elekta Infinity (Elekta AB, Stockholm, Sweden) operating at 6, 9, 12, and 15 MeV, and Varian TrueBeam (Varian Medical Systems, Palo Alto, CA) operating at 6, 9, 12, and 18 MeV. All measurements utilized a 10 cm × 10 cm^2^ electron applicator attachment. The electron applicators for Elekta and Varian machine differ in construction as is shown in Figure 1a‐d. The Varian electron applicator has support structures that have rounded edges with flat sides covering most of the b‐side as shown in Figure 1a. The total gap between the support structures on the a‐side is 13 cm and on the b‐side is 4.1 cm along the length of the applicator. In the case of Elekta, the support structures are cylindrical and placed toward the applicator edge as shown in Figure 1c,d. They have a larger gap between them: 18.5 cm on the a‐side and 14.5 cm on the b‐side. The design of the bottom of the applicator facing the patient is also different in both designs. Additionally, the base of the Varian electron applicator facing the patient is thicker (2.5 cm, see Figure 1a,b) and non‐detachable compared to Elekta's. In comparison, the Elekta applicator has a 1 cm thick insert and a holder that make up the bottom of the applicator.

Measurements were performed in a 3D water tank (Blue Phantom 2, Ion Beam Applications, Louvain‐la‐Neuve, Belgium), keeping the source‐to‐surface distance (SSD) at 100 cm. Profiles were measured at surface and d_max_, and at collimator (0°, 90°), and gantry angles (0°, 15°). Electron dose measurements in water were performed using an IBA EFD 3G‐pSi diode detector with an IBA RFD 3G‐pSi diode used as a reference.

As the water phantom could only accommodate a limited gantry angle, we used the IC Profiler (ICP) from Sun Nuclear to record profiles at angles >15°. The IC Profiler uses 2.9 mm wide ion chambers spaced 7 mm apart from each other on the diagonal and has 251 detectors and measurements possible along four cardinal directions and diagonals. The profiler was rotated 45° and the isocenter crosshair was placed at one of the diodes on the negative diagonal (‐D13) so that the profile outside the applicator field could be recorded.

When using the Elekta applicator, a maximum gantry angle of 39° was achieved using the same setup. In the case of Varian, due to the thicker base of the applicator, a gantry angle of 15° was the maximum that could be achieved. Measurements at d_max_ were recorded by placing solid water slices of required values and adjusting the SSD of the setup. Thin sheets of lead/lead apron were used to study how this scattered dose can be reduced and we believe this has significant clinical implications.

## 3 RESULTS

**FIGURE 2:**
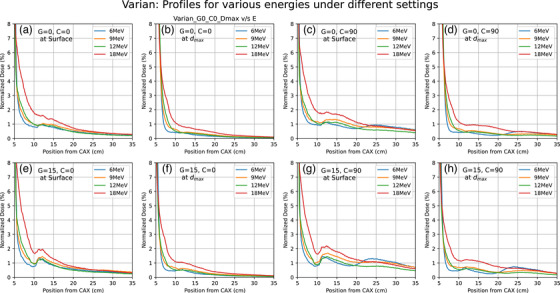
Profiles from using the Varian machine at different energies (6, 9, 12, and 18 MeV), depths (surface, d_max_), gantry (0,15), and collimator (0, 90) angles in the water phantom.

**FIGURE 3:**
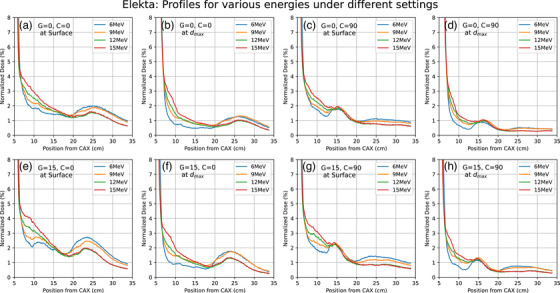
Profiles from using the Elekta machine at different energies (6, 9, 12, and 15 MeV), depths (surface, d_max_), gantry (0,15), and collimator (0, 90) angles in the water phantom.

**FIGURE 4:**
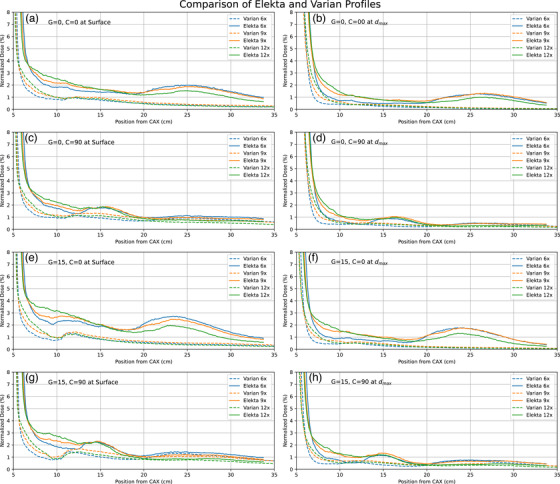
Comparison of Varian and Elekta profiles for different parameters.

Dose profiles were for various energies (6, 9, 12 MeV; 15 MeV—Elekta only and 18 MeV—Varian only), depths (surface, d_max_), collimator angles (0°, 90°) and gantry angles (0°, 15°) for both linacs are shown in Figures 2 and 3. It clearly shows secondary peaks outside the applicator field. Peaks are consistently more intense in profiles from Elekta compared to Varian. In the case of Varian, secondary peaks are found at 10−15 cm from the central axis and they become more pronounced on changing the collimator angle to 90° or on changing the gantry angle to 15°. For the Elekta measurements, the secondary peaks were observed between 20 and 30 cm from the central axis, and the peaks were observed to shift 15 cm toward CAX on the rotation of the collimator by 90°. No such shift was observed in profiles from Varian. In profiles from Elekta, there were no significant changes observed when the gantry angle was changed to 15°. As observed in Figure 1, the front and side construction of the Varian and Elekta applicators differ significantly in support beam placement and could be responsible for these positional variances between manufacturers. A comparison of Elekta and Varian profiles is shown in Figure 4 for various parameters.

Profiles recorded with ICP underwent comparison with those documented in the 3D water tank. In both instances, normalization to the CAX dose was implemented. The relative peak intensity and location demonstrated consistency with the absolute data derived from the 3D water tank.

**FIGURE 5:**
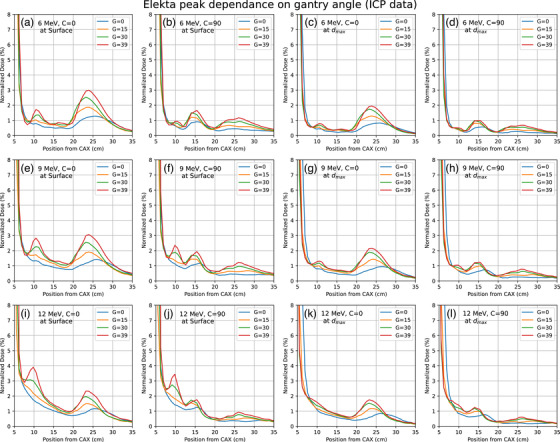
Effect of gantry angle on the secondary peaks from the Elekta device.

Since the measurements using the ICP were able to reproduce the initial data recorded using the water phantom, we used the ICP to study the gantry angle dependance of the secondary peaks. Figure 5 shows the profiles for different gantry angles under each setting. We observe that the peak positions become greater in relative magnitude and shift closer toward CAX as the gantry angle increases. Due to improved machine clearance, we were able to measure up to a 39° gantry angle on the Elekta machine. On the Varian machine, due to a thicker lower lip, we could not measure more than 15° due to clearance issues, the same as the absolute measurement performed on the water phantom. The peaks showed the same response on changes to collimator angle, depth, and energies. However, the peak intensity increased as we increased the gantry angle in all cases and all energies. We can observe a slight change in the peak position (2–4 cm) as we increase the gantry angle. As can be seen from the figure, the peaks shift toward the central axis as the gantry angle is increased for all energies and both collimator positions.

As previously suggested^21^, ^22^, ^23^, ^24^ that scatter electrons have very low energy and can be eliminated by thin sheet of materials like lead apron, lead sheet or Superflab. Our study found that a thin sheet (1 mm) of lead effectively eliminated the electron scatter dose. As an example, the comparison of 6 MeV profile where a 1 mm lead bolus was used is shown in Figure 7.

**FIGURE 6:**
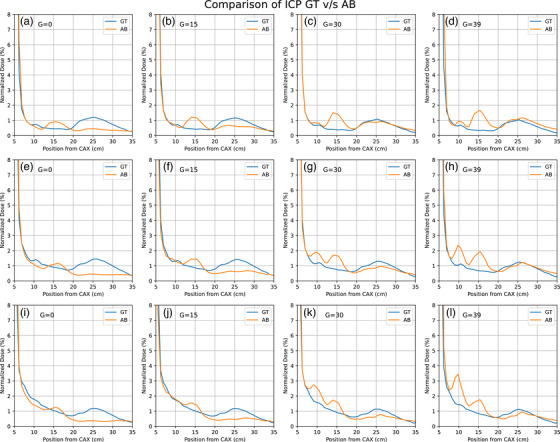
Profiles from the Elekta machine for different energies and Gantry angles showing the difference in measurement along the GT and AB direction. Plots A‐D are for 6 MeV, E‐H are for 10 MeV and I‐L are for 12 MeV. The collimator angle was 90° for all measurements shown in this figure.

The IC profiler was propped perpendicular to the electron applicator and all measurements were performed again to ensure that the peaks seen here are not a result of artifacts due to improper setup. For different gantry angles, the IC profiler was propped up on solid water slabs and taped to the electron applicator to ensure stability. The angle of the IC profiler was verified using a digital level. The results shown here were repeated under this setup. To ensure that these peaks were not a result of the electron applicator coming closer to the IC profiler, measurements were also performed along the GT direction where we found similar peaks. The comparison of results between the GT and AB direction are shown below in Figure 6.

**FIGURE 7:**
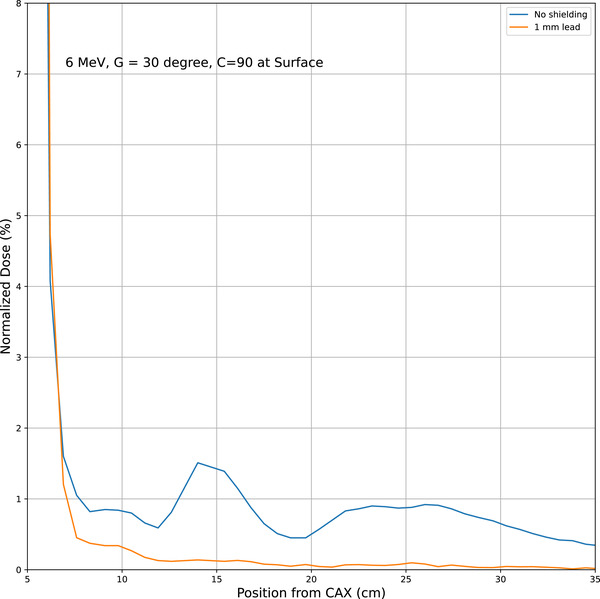
The effect of adding a 1 mm lead bolus on the profile.

## 4 DISCUSSIONS

The off‐axis profile measurements in a water phantom at surface and d_max_ for both Varian and Elekta machines for various electron energies indicates out‐of‐field electron scatter at significant distances (15−28 cm). The observation reveals that the location and intensity of the off‐axis radiation varies between Varian to Elekta, as well as with changes in the gantry and collimator angle. Profile measurements were performed using the IC profiler, validated against the water phantom measurements. Once verified, the IC profiler was used to record the profile for a broader range of gantry angles in the Elekta case, illustrating the changes in off‐axis dose is illustrated.

Electron beams are an essential tool in radiation therapy treatment and are commonly used to treat superficial targets and post‐surgery scars. The gantry angle is usually adjusted to target specific areas of the tumor while avoiding other structures, such as blood vessels or nerves, to deliver a high dose of radiation to the tumor while minimizing damage to surrounding healthy tissue. Besides the gantry angle, other parameters such as the energy of the electrons and the size and shape of the treatment field are also modulated to ensure appropriate radiation dose to the tumor. However, in most clinical scenarios, a non‐zero gantry angle setup is used.

Much attention is given to the head scatter from accelerators, which is usually <1%25, yet large angle electron scatters depositing a relatively higher dose (3%) often receive limited consideration as it is prudent to avoid any dose to normal tissue, understanding the magnitude and extent is critical, as demonstrated by this study. Off‐axis dose from electron beams have been studied extensively in the past. However, most of them are for older generation of the machines with different applicator designs.^7^, ^9^, ^10^, ^15^, ^19^ Even in recent studies, peripheral dose from electron beams is studied in a zero gantry angle setup,^17^, ^20^, ^26^, ^27^, ^28^ which does not accurately represent the setup used for clinical treatments. While Yeboah et al.^16^ studied the effect of obliquity of electron beam on peripheral dose, the data was recorded at one angle and used a Siemens Primus accelerator, which is no longer clinically available.^16^ This study presents the dependence of peripheral dose on gantry angle, an aspect not studied before. Our study is contemporary and contributes to existing knowledge, enabling more informed decisions in clinical scenarios. The angles studied were limited to 39° in the Elekta machine and 15° in the Varian machine due to clearance issues with the water phantom and the IC profiler. Additional insights could be garnered by Monte Carlo simulations, which is beyond the scope of the current work.

The current knowledge on electron scattering from modern linacs show us that dose peaks outside the applicator fields are still visible, however, this can be further reduced or eliminated altogether by thin sheet of lead or lead apron as has been suggested long back by Muller‐Runkel et al.^21^, ^22^ and recently by He et al.^17^ Other approaches for reducing scatter and electron contamination have been proposed, including body shielding, bolus and Aquaplast materials.^23^, ^24^, ^29^, ^30^ Given the scatter peaks are located far from the central axis (12−28 cm), this is often overlooked except in cases of clinically observed alopecia.^8^ In modern units, the scatter is still visible with 1%−3% depending upon the beam energy, gantry angle, and collimator angle. This can be attributed to the asymmetry of the applicators, which have wider openings in specific directions, thus causing more scatter.

## 5 CONCLUSION

The large angle electron scatter from applicators in modern Elekta (both in‐line and cross‐line) and Varian (cross‐line) machines presented in this work shows that the scatter's magnitude falls in the range of 1%−3% at a distance of 15−28 cm. The lack of symmetry in electron applicators leads to a difference in electron scatter from 0° and 90° collimators due to the variation in applicator openings. Wider openings result in higher scatter. Generally, the TrueBeam presents lower electron scatter (1%) compared to Elekta (3%). These long‐distance peaks from the treatment site can be mitigated by utilizing thin sheets of lead or lead aprons, Superflab, or other bolus materials, which helps reduce dose exposure to normal tissues.

## AUTHOR CONTRIBUTIONS

Concept: Indra J. Das; Data Collection: Bishwambhar Sengupta, Greg DeFillippo; Methodology, Writing Original Draft: Bishwambhar Sengupta, Poonam Yadav, Indra J. Das; Writing, review & Editing: Bishwambhar Sengupta, Poonam Yadav, Indra J. Das, James J. Sohn; Statistical Analysis: Bishwambhar Sengupta.

## CONFLICT OF INTEREST STATEMENT

The authors declare no conflicts of interest.
